# Glycometabolic profiles and pregnancy outcomes across pathophysiological subtypes of gestational diabetes mellitus

**DOI:** 10.3389/fendo.2026.1836589

**Published:** 2026-05-19

**Authors:** Junyou Su, Jing Luo, Xiaoting Huang, Yan Huang, Sumei Wang

**Affiliations:** 1 Department of Obstetrics, The First Affiliated Hospital of Guangxi Medical University, Nanning, China; 2 Department of Obstetrics, The Second Affiliated Hospital of Guangxi Medical University, Nanning, China

**Keywords:** gestational diabetes mellitus, glycometabolism, insulin resistance, pregnancy outcomes, subtypes

## Abstract

**Background:**

Gestational diabetes mellitus (GDM) exhibits profound pathophysiological heterogeneity, but current management fails to account for subtype-specific differences in clinical features and pregnancy prognoses. This study aimed to characterize the glycometabolic profiles and pregnancy outcomes of GDM subtypes defined by insulin resistance and β-cell function, and provide a foundation for precision medicine in GDM.

**Methods:**

In this 1:1 case−control study, 330 singleton women with GDM and 330 healthy singleton controls were enrolled. Using control−derived quartiles of HOMA−IR and HOMA−β, GDM patients were classified into four subtypes: insulin resistance (GDM−IR), insulin secretory defect (GDM−IS), combined defect (GDM−M), and unclassified (GDM−N). Clinical characteristics, glycometabolic parameters, and pregnancy outcomes were compared across groups, and multivariate logistic regression identified independent risk factors for adverse pregnancy outcomes.

**Results:**

Subtype distribution was: GDM−IR 36.4% (120/330), GDM−IS 22.7% (75/330), GDM−M 17.9% (59/330), GDM−N 23.0% (76/330). GDM−M showed the most severe glycometabolic derangements: highest first−trimester fasting glucose, all OGTT glucose values, HbA1c, insulin use rate (18.6%, 11/59), and poor glycemic control rate (22.0%, 13/59). It also had the highest rates of cesarean delivery (72.9%, 43/59), postpartum hemorrhage (8.5%, 5/59), preterm birth (18.6%, 11/59), macrosomia (17.0%, 10/59), and neonatal hypoglycemia (11.9%, 7/59) (all P < 0.05). Multivariate regression identified age (OR = 1.067, 95% CI: 1.028–1.108), pre−pregnancy BMI (OR = 1.073, 95% CI: 1.014–1.136), GDM−IS (OR = 2.013, 95% CI: 1.134–3.574), GDM−M (OR = 8.417, 95% CI: 2.661–26.627), and GDM−N (OR = 1.831, 95% CI: 1.076–3.115) as independent risk factors for adverse pregnancy outcomes, with GDM−M conferring the highest risk.

**Conclusions:**

GDM subtypes defined by insulin resistance and β−cell function exhibit marked heterogeneity in glycometabolic profiles and pregnancy outcomes. The combined defect subtype (GDM−M) carries the highest risk of adverse maternal−neonatal events and may warrant the most intensive monitoring. These findings support pathophysiological subtyping for risk stratification, but causal inference is limited by the observational, single−center design; validation in multicenter, multi−ethnic prospective cohorts is required.

## Introduction

Gestational diabetes mellitus (GDM) is one of the most prevalent pregnancy complications worldwide, affecting approximately 14% of all pregnancies, with prevalence reaching as high as 27.6% in some regions (1, 2). Driven by shifts in national fertility policies, a rising proportion of pregnant women of advanced maternal age, and widespread lifestyle changes, the incidence of GDM in China has increased markedly, with a reported prevalence of up to 24.24% in some regions (3). The pathogenesis of GDM is multifactorial and complex, which is currently recognized to be closely associated with insulin resistance, impaired insulin secretion, genetic predisposition, and environmental factors (4). GDM not only elevates the risk of short-term adverse pregnancy outcomes including preeclampsia, preterm birth, cesarean delivery, and macrosomia, but is also strongly linked to long-term maternal complications such as type 2 diabetes mellitus, metabolic syndrome, and cardiovascular disease, as well as adverse long-term offspring outcomes including obesity, impaired glucose tolerance, metabolic syndrome, and visuomotor dysfunction (5, 6). To date, there are no validated effective preventive measures for GDM, and clinical management mainly focuses on glycemic control during pregnancy via dietary modification, regular physical activity, and pharmacological interventions (e.g., metformin, insulin) (2). In conventional clinical practice, a standardized, one-size-fits-all approach to diagnosis and management is routinely used for patients with GDM, with treatment adjustments based on dynamic blood glucose monitoring. Although stepwise escalation of lifestyle modification and timely pharmacological intervention can mitigate a portion of these adverse risks (4), the overall therapeutic efficacy remains suboptimal.

The core pathophysiology of GDM is the failure of pancreatic β-cell insulin secretion to compensate for physiological pregnancy-induced insulin resistance (5), and GDM can be stratified into pathophysiologically distinct subtypes (insulin resistance-predominant, insulin secretion-deficient, and combined) with differential long-term metabolic risks (7). However, most prior studies have disproportionately focused on these long-term outcomes, with limited systematic head-to-head comparisons of short-term glycometabolic phenotypes, clinical intervention requirements, and comprehensive perinatal outcomes across subtypes. Critically, existing subtyping frameworks use cut-off values derived from non-pregnant or heterogeneous cohorts, which fail to account for gestational physiological adaptations in insulin sensitivity and β-cell function. To address these unmet gaps, we hypothesized that GDM subtypes defined by homeostasis model assessment of insulin resistance (HOMA-IR) and β-cell function (HOMA-β) (using cut-offs from a contemporaneous, geographically matched cohort of pregnant women with normal glucose tolerance) would exhibit marked heterogeneity in glycometabolic profiles and perinatal outcomes, and specifically that the combined defect subtype would be associated with the most severe glycemic dysregulation and highest risk of adverse maternal and neonatal events. We therefore stratified 330 GDM patients into four pathophysiological subtypes, systematically compared their clinical and metabolic characteristics, and identified independent risk factors for composite adverse perinatal outcomes, to provide evidence-based support for subtype-specific precision management of GDM.

## Materials and methods

### Study subjects

This study employed a 1:1 frequency-matched case-control design. A total of 330 singleton pregnant women with GDM who received regular antenatal care and delivered at The Second Affiliated Hospital of Guangxi Medical University between January 2024 and September 2025 were enrolled as the study group. An additional 330 oral glucose tolerance test (OGTT)-confirmed normal glucose tolerance (NGT) singleton pregnant women who received antenatal care during the same period were selected as the control group, frequency-matched for singleton pregnancy status and enrollment period at the group level; no individual matching on age, pre-pregnancy body mass index (BMI), or other covariates was performed. Adjustment for potential confounders was performed in multivariate regression.

All participants (both GDM and control groups) underwent standardized 75g OGTT at 24–28 weeks of gestation using identical laboratory protocols. Inclusion Criteria: ① GDM was diagnosed in accordance with the “Chinese Guidelines for the Diagnosis and Treatment of Hyperglycemia in Pregnancy (2022)” (8): fasting plasma glucose (FPG) ≥5.1 mmol/L, or 1-hour postprandial plasma glucose (1-h PPG) ≥10.0 mmol/L, or 2-hour postprandial plasma glucose (2-h PPG) ≥8.5 mmol/L; the control group was defined as having all three OGTT glucose values below the above diagnostic thresholds; ② Singleton pregnancy; ③ Complete clinical and laboratory data. Exclusion Criteria: ① Pre-pregnancy diagnosis of chronic diseases, including diabetes mellitus, hypertension, heart disease, and hepatic or renal dysfunction; ② Multiple pregnancy or ectopic pregnancy; ③ Complicated with pregnancy-specific disorders including gestational hypertension, placental abruption, and placenta previa; ④ Mental illness or poor treatment compliance.

Written informed consent was obtained from all participants prior to enrollment. This study was approved by the Ethics Committee of The Second Affiliated Hospital of Guangxi Medical University [Approval No. 2026-KYL (010)].

### GDM subtyping criteria

GDM subtyping was performed according to the method previously described by Lee et al. (9), which anchors cut−off values to the quartiles of insulin sensitivity and insulin secretory function derived from pregnant women with NGT. This approach uses control−derived quartiles to reflect the physiological distribution of insulin sensitivity and β−cell function in a healthy pregnant population and has been validated in prior GDM subtyping studies. In this study, the cut-off values were defined according to the quartiles of HOMA-IR and HOMA-β from the control group. HOMA-IR was used to evaluate the degree of insulin resistance (higher value indicates more severe insulin resistance and lower insulin sensitivity), calculated using the following formula: HOMA-IR = FPG (mmol/L) × fasting insulin (FINS, μU/mL)/22.5 (10). HOMA-β was applied to assess pancreatic β-cell insulin secretory capacity (higher value indicates better secretory function), calculated using the following formula: HOMA-β = 20 × FINS/(FPG – 3.5) (11). The quartiles (P25, P75) of the control group were 1.49 and 2.64 for HOMA-IR, and 242.60 and 455.53 for HOMA-β. Based on these cut-off values, women with GDM were stratified into four subtypes: Insulin resistance subtype (GDM-IR): HOMA-IR > 2.64 and HOMA-β ≥ 242.60; Insulin secretory defect subtype (GDM-IS): HOMA-β < 242.60 and HOMA-IR ≤ 2.64; Combined defect subtype (GDM-M): HOMA-IR > 2.64 and HOMA-β < 242.60; Unclassified subtype (GDM-N): HOMA-IR ≤ 2.64 and HOMA-β ≥ 242.60.

### Outcome measures

The following data were collected from the hospital electronic medical record system and laboratory information system: (1) General clinical data: maternal age, proportion of advanced maternal age (≥35 years), gravidity, parity, and BMI; (2) Glycometabolic parameters: first-trimester FPG (measured at 6–13 + 6 weeks of gestation); FPG, FINS, 1-h PPG and 2-h PPG were all measured on the same day as the diagnostic OGTT (24–28 weeks of gestation) using standardized, quality-controlled laboratory assays; glycated hemoglobin A1c (HbA1c); insulin therapy status; and proportion of participants with poor glycemic control (defined as HbA1c ≥6.0% measured at 32–34 weeks of gestation, before delivery) (8, 12); (3) Pregnancy outcomes: maternal outcomes (including premature rupture of membranes, fetal distress, gestational age at delivery, delivery mode, and postpartum hemorrhage) and neonatal outcomes (including birth weight, macrosomia [defined as birth weight ≥4000g], neonatal hypoglycemia [defined as blood glucose ≤2.6 mmol/L] (13), neonatal hyperbilirubinemia, and neonatal asphyxia); (4) Adverse pregnancy outcome: the occurrence of any of the following: fetal distress, postpartum hemorrhage, preterm birth, cesarean delivery, macrosomia, neonatal asphyxia, neonatal hypoglycemia, or neonatal hyperbilirubinemia.

### Statistical analysis

All statistical analyses were performed using SPSS version 26.0 software (IBM Corp., Armonk, NY, USA). Normally distributed continuous variables were presented as mean ± standard deviation (*χ¯ ± s*). One-way analysis of variance (ANOVA) was used for intergroup comparisons, with *post-hoc* pairwise comparisons performed using the LSD t-test. Categorical variables were expressed as number (percentage), and comparisons between groups were conducted using the chi-square (*χ²*) test or Fisher’s exact test, as appropriate. The composite adverse pregnancy outcome was defined as the occurrence of any of the following: fetal distress, postpartum hemorrhage, preterm birth, cesarean delivery, macrosomia, neonatal asphyxia, neonatal hypoglycemia, or neonatal hyperbilirubinemia. This composite was coded as a binary variable (0 = no event, 1 = at least one event). Women could experience multiple events, but the composite outcome was coded as 1 if any event occurred. Multivariate logistic regression analysis was performed with composite adverse pregnancy outcome as the dependent variable. Covariates were selected *a priori* based on established clinical risk factors for adverse perinatal outcomes in GDM, including maternal age, pre-pregnancy BMI, gravidity, parity, and gestational age at delivery. Collinearity between covariates was assessed using variance inflation factor (VIF) analysis; all VIF values were <2.0, indicating no significant multicollinearity. A two-sided *P*-value < 0.05 was considered statistically significant.

## Results

### Comparison of general clinical characteristics across GDM subtypes

Among 330 women with GDM, the distribution of pathophysiological subtypes was as follows: GDM-IR 36.36% (120/330), GDM-IS 22.73% (75/330), GDM-M 17.88% (59/330), GDM-N 23.03% (76/330). All GDM subtypes presented elevated maternal age and a higher proportion of advanced maternal age relative to normoglycemic controls, with the GDM-M subgroup exhibiting the oldest mean maternal age among all GDM subsets. Pre-pregnancy BMI was significantly increased in GDM-IR and GDM-M compared with the control, GDM-IS and GDM-N groups. Gestational age at delivery was the lowest in the GDM-M subgroup, while that of the GDM-N subgroup was comparable to controls. Gravidity and parity showed no intergroup differences across the entire study cohort (**Table 1**).

**Table 1:** Comparison of general clinical characteristics across GDM subtypes [n (%)/*χ¯ ± s*].

Group	Control (n=330)	GDM-IR (n=120)	GDM-IS (n=75)	GDM-M (n=59)	GDM-N (n=76)	*F/χ²*	*P*
Age (years)	30.78 ± 4.58	32.55 ± 4.32^a^	32.69 ± 4.82^a^	33.53 ± 4.36^a^	31.72 ± 4.34^a^,^d^	7.831	<0.001
Advanced maternal age	80 (24.24)	66 (55.00)^a^	48 (64.00)^a^	29 (49.15)^a^	42 (55.26)^a^	72.730	<0.001
Pre-pregnancy BMI (kg/m²)	26.57 ± 3.21	29.02 ± 3.19^a^	25.88 ± 2.79^b^	28.62 ± 3.57^a^,^c^	25.86 ± 2.49^b^,^d^	22.909	<0.001
Gravidity	2.22 ± 1.30	2.38 ± 1.23	2.32 ± 1.43	2.31 ± 1.56	2.34 ± 1.24	0.672	0.511
Parity	1.62 ± 0.71	1.69 ± 0.75	1.66 ± 0.74	1.78 ± 0.77	1.67 ± 0.74	0.652	0.626
Gestational age at delivery (weeks)	38.62 ± 1.56	38.36 ± 1.63	38.17 ± 1.73 ^a^	37.73 ± 2.23 ^a^,^b^,^c^	38.37 ± 1.21^d^	4.371	0.002

^a^ vs. Control group, *P* < 0.05; ^b^ vs. GDM-IR group, *P* < 0.05; ^c^ vs. GDM-IS group, *P* < 0.05; ^d^ vs. GDM-M group, *P* < 0.05.

### Comparison of glycometabolic parameters across GDM subtypes

GDM-M was characterized by the most profound glycometabolic impairment, manifesting as the highest first-trimester FPG and all OGTT glucose measurements across all study groups. First-trimester FPG levels were comparable between the GDM-N subgroup and normoglycemic controls; OGTT FPG was lower in GDM-N than in other GDM subtypes, whereas 1-hour and 2-h PPG concentrations showed no substantial intergroup differences among GDM-N, GDM-IR, and GDM-IS.

FINS levels and HOMA-IR were markedly elevated in GDM-IR and GDM-M. HOMA-β was highest in GDM-N and intermediate in GDM-IR, with significant reductions in GDM-IS and GDM-M relative to controls and other GDM subsets.

With respect to clinical management, GDM-M demonstrated the greatest therapeutic demand secondary to severe glycemic dysregulation, with the highest rates of insulin therapy (18.64%, 11/59) and suboptimal glycemic control (22.03%, 13/59), followed by the GDM-IR subgroup (**Table 2**).

**Table 2:** Comparison of glycometabolic parameters across GDM subtypes [n (%)/*χ¯ ± s*].

Group	Control (n=330)	GDM-IR (n=120)	GDM-IS (n=75)	GDM-M (n=59)	GDM-N (n=76)	*F/χ²*	*P*
FPG (first trimester, mmol/L)	4.49 ± 0.32	4.60 ± 0.36^a^	4.70 ± 0.61^a^	5.09 ± 0.59^a^,^b^,^c^	4.41 ± 0.28^b^,^c^,^d^	34.592	<0.001
FPG (OGTT, mmol/L)	4.21 ± 0.29	4.54 ± 0.42^a^	4.49 ± 0.27^a^	5.47 ± 0.62^a^,^b^,^c^	4.03 ± 0.23^a^,^b^,^c^,^d^	187.300	<0.001
1-h PPG (OGTT, mmol/L)	7.42 ± 1.33	10.28 ± 1.42^a^	10.28 ± 1.55^a^	11.63 ± 2.09^a^,^b^,^c^	10.27 ± 1.94^a^,^d^	202.252	<0.001
2-h PPG (OGTT, mmol/L)	6.19 ± 1.15	8.85 ± 1.47^a^	9.16 ± 1.59^a^	10.51 ± 1.99^a^,^b^,^c^	8.82 ± 1.12^a^,^d^	221.615	<0.001
FINS (μU/mL)	11.59 ± 4.87	20.60 ± 7.33^a^	8.88 ± 1.99^a^,^b^	16.99 ± 4.31^a^,^b^,^c^	10.53 ± 2.7^b^,^c^	104.291	<0.001
HOMA-IR	2.20 ± 1.04	4.21 ± 1.81^a^	1.78 ± 0.46^a^,^b^	4.17 ± 1.34^a^,^c^	1.90 ± 0.49^a^,^b^,^d^	110.871	<0.001
HOMA-β	368.95 ± 183.94	491.72 ± 394.89^a^	187.04 ± 40.46^a^,^b^	182.96 ± 45.97^a^,^b^	540.47 ± 549.46^a^,^c^,^d^	26.637	<0.001
HbA1c (%)	–	5.69 ± 0.48	5.56 ± 0.38	5.83 ± 0.63^c^	5.31 ± 0.36^b^,^c^,^d^	19.533	<0.001
Insulin therapy	0 (0.00)	4 (3.33)^a^	1 (1.33)	11 (18.64)^a^,^b^,^c^	1 (1.32)^d^	49.845	<0.001
Poor glycemic control (HbA1c ≥6.0%)	0 (0.00)	19 (15.83)^a^	6 (8.00)^a^,^b^	13 (22.03)^a^,^c^	3 (3.95)^a^,^b^,^d^	58.831	<0.001

^a^ vs. Control group, *P* < 0.05; ^b^ vs. GDM-IR group, *P* < 0.05; ^c^ vs. GDM-IS group, *P* < 0.05; ^d^ vs. GDM-M group, *P* < 0.05.

### Comparison of pregnancy outcomes across GDM subtypes

Regarding maternal perinatal outcomes, fetal distress was more prevalent across all GDM subtypes relative to normoglycemic controls, with the highest incidence observed in the GDM−IS subgroup. The GDM−M subtype demonstrated the highest rates of cesarean delivery (72.88%, 43/59), postpartum hemorrhage (8.47%, 5/59) and preterm birth (18.64%, 11/59); additionally, the rate of cesarean delivery was significantly higher in the GDM−IR subgroup than in controls. No intergroup differences were detected in the incidence of premature rupture of membranes or neonatal asphyxia.

For neonatal outcomes, the GDM−M subtype exhibited the highest incidences of macrosomia (16.95%, 10/59), elevated mean birth weight (3281.10 ± 706.63 g) and neonatal hypoglycemia (11.86%, 7/59). Neonatal hyperbilirubinemia was more common in the GDM−IS and GDM−M subgroups compared with controls, while all adverse neonatal outcomes in the GDM−N subgroup were comparable to those in the normoglycemic control group (**Table 3**).

**Table 3:** Comparison of pregnancy outcomes across GDM subtypes [n (%)/*χ¯ ± s*].

Group	Control (n=330)	GDM-IR (n=120)	GDM-IS (n=75)	GDM-M (n=59)	GDM-N (n=76)	*F/χ²*	*P*
Premature rupture of membranes	73 (22.12)	22 (18.33)	11 (14.67)	3 (5.08)^a^,^b^	18 (23.68)^d^	8.323	0.080
Fetal distress	26 (7.88)	17 (14.17)^a^	16 (21.33)^a^	10 (16.95)^a^	7 (9.21)^c^	14.312	0.006
Cesarean delivery	107 (32.42)	60 (50.00)^a^	28 (37.33)	43 (72.88)^a^,^b^,^c^	34 (44.74)^d^	39.623	<0.001
Postpartum hemorrhage	7 (2.12)	2 (1.67)	1 (1.33)	5 (8.47)^a^,^b^,^c^	0 (0.00)^d^	8.462	0.042*
Preterm birth	20 (6.06)	17 (14.17)^a^	11 (14.67)^a^	11 (18.64)^a^	5 (6.58)^b^,^c^,^d^	16.074	0.003
Macrosomia	6 (1.82)	7 (5.83)	1 (1.33)	10 (16.95)^a^,^b^,^c^	0 (0.00)^d^	26.125	<0.001*
Neonatal birth weight (g)	3127.08 ± 419.35	3214.58 ± 513.52	3056.33 ± 507.84^b^	3281.10 ± 706.63 ^a^,^c^	3087.11 ± 377.11^d^	2.904	0.021
Neonatal asphyxia	6 (1.82)	4 (3.33)	4 (5.33)	1 (1.69)	0 (0.00)	5.347	0.183*
Neonatal hypoglycemia	5 (1.51)	4 (3.33)^a^	2 (2.67)	7 (11.86)^a^,^b^,^c^	1 (1.32)^d^	13.627	0.004*
Neonatal hyperbilirubinemia	52 (15.76)	28 (23.33)	20 (26.67)^a^	20 (33.90)^a^	15 (19.74)	13.442	0.009

^a^ vs. Control group, *P* < 0.05; ^b^ vs. GDM-IR group, *P* < 0.05; ^c^ vs. GDM-IS group, *P* < 0.05; ^d^ vs. GDM-M group, *P* < 0.05; * Fisher’s exact test.

### Risk factor analysis for adverse pregnancy outcomes in GDM

The odds ratios (ORs) presented in **Table 4** are adjusted for maternal age, pre−pregnancy BMI, gravidity, parity, and gestational age at delivery. Multivariate logistic regression analysis was conducted with the occurrence of composite adverse pregnancy outcome (including fetal distress, postpartum hemorrhage, preterm birth, cesarean delivery, macrosomia, neonatal asphyxia, neonatal hypoglycemia, and neonatal hyperbilirubinemia) as the dependent variable. The results showed that age (OR = 1.067, 95% CI: 1.028–1.108), pre-pregnancy BMI (OR = 1.073, 95% CI: 1.014–1.136), GDM-IS (OR = 2.013, 95% CI: 1.134–3.574), GDM-M (OR = 8.417, 95% CI: 2.661–26.627), and GDM-N (OR = 1.831, 95% CI: 1.076–3.115) were independent risk factors for adverse pregnancy outcomes (all *P* < 0.05). GDM-IR (OR = 1.481, 95% CI: 0.858–2.555) was not independently associated with adverse pregnancy outcomes (*P* > 0.05) (**Table 4**).

**Table 4:** Multivariate logistic regression analysis of independent risk factors for adverse pregnancy outcomes in GDM.

Variable	B	Standard error	P value	OR	95% CI
Age	0.065	0.019	<0.001	1.067	1.028 – 1.108
Pre-pregnancy BMI	0.071	0.029	0.015	1.073	1.014 – 1.136
GDM-IR	0.393	0.278	0.158	1.481	0.858 – 2.555
GDM-IS	0.700	0.293	0.017	2.013	1.134 – 3.574
GDM-M	2.130	0.588	<0.001	8.417	2.661 – 26.627
GDM-N	0.605	0.271	0.026	1.831	1.076 – 3.115

## Discussion

As the most prevalent metabolic complication of pregnancy, the global incidence of GDM continues to rise. In this study, we stratified women with GDM into pathophysiological subtypes based on insulin resistance and β-cell function, and confirmed significant heterogeneity in clinical characteristics, glycometabolic profiles, and pregnancy outcomes across distinct subtypes. Notably, the GDM-M subtype (concurrent insulin resistance and insulin secretory defect) was associated with the most severe glycemic dysregulation, the greatest difficulty in clinical management, and the highest risk of adverse maternal and neonatal outcomes. However, given the relatively small sample size of some subgroups (especially GDM−M) and potential residual confounding, effect sizes in these subgroups should be interpreted with caution. Nevertheless, metabolic subtyping may provide a useful reference for individualized risk assessment in clinical practice.

In our study cohort, the GDM-IR subtype was the most prevalent, accounting for 36.4% of the overall GDM population, indicating that insulin resistance-predominant GDM is the most common subtype among pregnant women in this region. Women with this subtype had a significantly higher pre-pregnancy BMI, which is closely linked to obesity-induced insulin resistance. In the obese state, adipokines such as adiponectin and leptin secreted by adipose tissue can interfere with the insulin signaling pathway, leading to reduced insulin sensitivity in peripheral tissues (14). In addition, maternal age and the proportion of women of advanced maternal age were significantly elevated in all GDM subtypes, consistent with advanced maternal age being a well-established risk factor for GDM. The GDM-M group had the oldest mean age, which may be explained by the gradual decline in pancreatic β-cell functional reserve with increasing age. When confronted with the physiological insulin resistance of pregnancy, these women are unable to mount an adequate compensatory insulin secretory response, resulting in a combined defect where both factors synergistically increase the risk of GDM. Multivariate logistic regression analysis confirmed that age and pre-pregnancy BMI were independent risk factors for adverse pregnancy outcomes in GDM. This highlights the clinical need for early assessment of islet function and insulin resistance in high-risk pregnant women (e.g., those of advanced maternal age or with pre-pregnancy obesity), to enable proactive intervention and reduce the risk of adverse perinatal outcomes.

The analysis of glycometabolic characteristics further elucidated the fundamental pathophysiological differences between the subtypes. The GDM-M group displayed the most pronounced hyperglycemic phenotype, with significantly higher first-trimester FPG, glucose levels at all OGTT time points, HbA1c levels, rates of insulin therapy, and rates of poor glycemic control compared with all other GDM subtypes. This finding indicates that women with GDM and concurrent insulin resistance and β-cell dysfunction have a more profound impairment of glucose homeostasis, and achieving optimal glycemic control remains challenging even after insulin initiation. The underlying mechanisms for this are likely twofold: First, peripheral insulin resistance increases hepatic gluconeogenesis and impairs glucose uptake in peripheral tissues, leading to elevated basal and postprandial glucose levels (15); Second, insufficient compensatory β-cell function fails to meet the increased insulin demand required to counteract hyperglycemia (16). In contrast, although the GDM-IR group exhibited severe insulin resistance (as indicated by the highest HOMA-IR), their β-cell function showed compensatory enhancement (the second-highest HOMA-β), which partially offset the insulin resistance and resulted in less pronounced hyperglycemia than that observed in the GDM-M group. The GDM-IS subtype, characterized by isolated insulin secretory defect, had mild insulin resistance but still presented with significantly elevated postprandial glucose levels, indicating marked glycemic variability. The GDM-N group had a metabolic profile closest to that of healthy pregnant women, with preserved insulin secretion, only mild postprandial hyperglycemia, and the best glycemic control.

In terms of pregnancy outcomes, the GDM-M group had the most prominent risk of adverse maternal and neonatal events, with significantly elevated rates of cesarean delivery, postpartum hemorrhage, preterm birth, macrosomia, and neonatal hypoglycemia. Multivariate logistic regression analysis further confirmed that GDM-M was the strongest independent risk factor for adverse pregnancy outcomes (OR = 8.417). This finding is most likely attributable to the poor glycemic control and chronic intrauterine hyperglycemic exposure in this group. Persistent maternal hyperglycemia can induce vascular endothelial injury in the placenta, leading to impaired placental perfusion and an increased risk of fetal distress (17). Simultaneously, maternal hyperglycemia crosses the placenta and induces fetal hyperglycemia, which stimulates excessive insulin secretion by fetal pancreatic β-cells and causes fetal hyperinsulinemia. This in turn drives excessive fetal growth and macrosomia, and after birth, the abrupt interruption of maternal glucose supply in the context of fetal hyperinsulinemia results in neonatal hypoglycemia (18–20). Furthermore, poor glycemic control increases the risk of cesarean delivery and postpartum hemorrhage, which are frequently necessitated by macrosomia or non-reassuring fetal status. Adverse outcomes in the GDM-IR group were mainly characterized by an increased cesarean delivery rate, which was associated with the high pre-pregnancy BMI and mildly elevated macrosomia incidence in this group. Interestingly, although the GDM-IR group showed a trend toward increased adverse pregnancy outcomes in univariate analysis, it was not an independent risk factor in the multivariate model. Two plausible mechanisms account for this result. First, the adverse effect of GDM−IR was largely mediated by pre−pregnancy BMI, an independent risk factor in the adjusted model, which attenuated the independent contribution of the IR subtype. Second, patients with GDM−IR exhibited preserved (even supranormal) β−cell function (mean HOMA−β: 491.72 vs. 368.95 in controls), mounting a compensatory insulin secretory response that reduced hyperglycemic severity and lowered risks of preterm birth, macrosomia, and neonatal hypoglycemia compared with the GDM−M subgroup. Furthermore, the limited sample size of the GDM−IR subgroup (n=120) may have resulted in moderate statistical power, constraining detection of the modest independent effect (OR≈1.5). The GDM-IS group was mainly characterized by higher rates of fetal distress and neonatal hyperbilirubinemia, which may be related to the marked postprandial glycemic fluctuations caused by insufficient insulin secretion. Such glycemic variability can increase the risk of intrauterine fetal hypoxia, and may also affect the maturation of fetal liver enzymes, thereby interfering with bilirubin metabolism (21). Although the GDM-N group achieved good glycemic control, it was still identified as an independent risk factor for adverse pregnancy outcomes in the regression model. This suggests that even mild, well-compensated GDM may involve other unrecognized pathophysiological pathways that contribute to adverse outcomes such as fetal distress; thus, standardized perinatal monitoring is still warranted for this subtype.

This GDM subtyping strategy based on fasting insulin and glucose has clear potential for translation into routine obstetric care. As fasting insulin and glucose measurements are already standard components of prenatal metabolic screening in clinical practice, this subtyping approach does not require additional invasive testing or complex laboratory workflows. Identification of high-risk subtypes such as GDM-M could enable clinicians to implement targeted interventions, including intensified glycemic surveillance, personalized dietary and lifestyle counseling, and early obstetric planning, thereby facilitating precision prevention and stratified management of GDM. Future prospective studies are warranted to validate the clinical utility of this subtyping approach in large-scale routine care settings.

### Limitations

Several limitations should be acknowledged. First, HOMA−IR and HOMA−β are indirect measures of insulin resistance and β−cell function. Although widely used in GDM research, their accuracy depends on the timing of blood sampling (22–24), and no universally standardized cut−offs exist. HOMA−IR and HOMA−β cut−offs were derived from quartiles of a contemporaneous, geographically matched healthy control group. Although we followed a previously validated method, some degree of misclassification may occur, particularly for patients with values near the thresholds. We did not perform sensitivity analyses using alternative cut−offs, as such analyses would require independent validation cohorts and standardized protocols. These thresholds are population−specific and may not be directly transferable to other ethnic groups or geographic regions due to known differences in insulin sensitivity, β−cell reserve, and assay methods. Second, multiple statistical comparisons across GDM subtypes and various pregnancy outcomes were conducted without adjustment for multiple comparisons, which may increase the risk of inflated Type I error. Third, this single−center study with a sample size that is modest for certain subgroup (especially GDM-M) analyses may yield insufficient statistical power for some comparisons. Accordingly, effect sizes in these smaller subgroups should be interpreted with caution. Additionally, potential residual confounding cannot be excluded, as important factors including gestational weight gain, smoking status, socioeconomic status, and prior obstetric history were not adjusted for in the analyses. Moreover, the use of a broad composite outcome incorporating heterogeneous events including cesarean delivery, macrosomia, and neonatal hypoglycemia may potentially obscure subtype−specific risk patterns, although individual outcomes were separately analyzed in **Table 3** to reduce this concern. Finally, long−term follow−up data on maternal and offspring metabolic outcomes were not collected.

### Future directions

Future studies should incorporate continuous glucose monitoring, adopt multicenter large−scale cohort designs, and include long−term follow−up to further validate the clinical utility of GDM subtyping. Specifically, large−scale multicenter studies should perform sensitivity analyses using alternative HOMA−IR and HOMA−β cut−offs—such as population−specific percentiles or receiver operating characteristic (ROC)−derived thresholds—to assess the stability of subtype classification and its association with adverse pregnancy outcomes. Advancing pathophysiology−based classification strategies will be critical for enabling precise prevention and treatment of GDM, thereby improving both short− and long−term outcomes for mothers and their offspring.

## Conclusions

In conclusion, significant heterogeneity exists in clinical characteristics, glycometabolic profiles, clinical intervention requirements, and pregnancy outcomes across distinct GDM subtypes. The GDM-M subtype is associated with the most severe glycemic dysregulation, the highest clinical intervention demand, and the poorest pregnancy outcomes; therefore, it may warrant prioritization for targeted clinical monitoring and intensified intervention, with early identification recommended. For the GDM-IR subtype, clinical management should focus on pre-pregnancy weight control and gestational weight gain management to improve insulin sensitivity. For the GDM-IS subtype, close monitoring of glycemic variability and exploration of interventions to preserve β-cell function are warranted. Although the overall risk of the GDM-N subtype is relatively low, standardized perinatal management is still necessary. These findings support pathophysiological subtyping for risk stratification, but causal inference is limited by the observational, single−center design; validation in multicenter, multi−ethnic prospective cohorts is required.

## Data Availability

The original contributions presented in the study are included in the article/**Supplementary Material**. Further inquiries can be directed to the corresponding authors.

## References

[1] Hod M Kapur A McIntyre HD FIGO Working Group on Hyperglycemia in Pregnancy FIGO Pregnancy and Prevention of early NCD Committee . Evidence in support of the International Association of Diabetes in Pregnancy study groups' criteria for diagnosing gestational diabetes mellitus worldwide in 2019. Am J Obstet Gynecol. (2019) 221:109–16. doi: 10.1016/j.ajog.2019.01.206. PMID: 30682358

[2] Sweeting A Hannah W Backman H Catalano P Feghali M Herman WH . Epidemiology and management of gestational diabetes. Lancet. (2024) 404:175–92. doi: 10.1016/S0140-6736(24)00825-0. PMID: 38909620

[3] Juan J Yang H . Prevalence, prevention, and lifestyle intervention of gestational diabetes mellitus in China. Int J Environ Res Public Health. (2020) 17:9517. doi: 10.3390/ijerph17249517. PMID: 33353136 PMC7766930

[4] Farrar D . Hyperglycemia in pregnancy: prevalence, impact, and management challenges. Int J Womens Health. (2016) 8:519–27. doi: 10.2147/IJWH.S102117. PMID: 27703397 PMC5036767

[5] Hivert MF Backman H Benhalima K Catalano P Desoye G Immanuel J . Pathophysiology from preconception, during pregnancy, and beyond. Lancet. (2024) 404:158–74. doi: 10.1016/S0140-6736(24)00827-4. PMID: 38909619

[6] McIntyre HD Catalano P Zhang C Desoye G Mathiesen ER Damm P . Gestational diabetes mellitus. Nat Rev Dis Primers. (2019) 5:47. doi: 10.1038/s41572-019-0098-8. PMID: 31296866

[7] Osmulski ME Yu Y Kuang A Josefson JL Hivert MF Scholtens DM . Subtypes of gestational diabetes mellitus are differentially associated with newborn and childhood metabolic outcomes. Diabetes Care. (2025) 48:390–9. doi: 10.2337/dc24-1735. PMID: 39787502 PMC11870284

[8] Wang C Juan J Yang H Maternal-Fetal Medicine Committee Chinese Society of Obstetrics and Gynecology Chinese Medical Association . A summary of Chinese guidelines on diagnosis and management of hyperglycemia in pregnancy (2022). Matern Fetal Med. (2023) 5:4–8. doi: 10.1097/FM9.0000000000000181. PMID: 40433235 PMC12106219

[9] Lee K Kuang A Bain JR Hayes MG Muehlbauer MJ Ilkayeva OR . Metabolomic and genetic architecture of gestational diabetes subtypes. Diabetologia. (2024) 67:895–907. doi: 10.1007/s00125-024-06110-x. PMID: 38367033 PMC12560237

[10] Alptekin H Çizmecioğlu A Işık H Cengiz T Yildiz M Iyisoy MS . Predicting gestational diabetes mellitus during the first trimester using anthropometric measurements and HOMA-IR. J Endocrinol Invest. (2016) 39:577–83. doi: 10.1007/s40618-015-0427-z. PMID: 26754418

[11] Shen Y Zheng Y Su Y Jiang S Ma X Hu J . Insulin sensitivity, β cell function, and adverse pregnancy outcomes in women with gestational diabetes. Chin Med J (Engl). (2022) 135:2541–6. doi: 10.1097/CM9.0000000000002337. PMID: 36583917 PMC9944688

[12] ACOG Practice Bulletin No. 190 . Gestational diabetes mellitus. Obstet Gynecol. (2018) 131:e49–64. doi: 10.1097/AOG.0000000000002501. PMID: 29370047

[13] Wight N Marinelli KA Academy of Breastfeeding Medicine . ABM clinical protocol #1: guidelines for blood glucose monitoring and treatment of hypoglycemia in term and late-preterm neonates, revised 2014. Breastfeed Med. (2014) 9:173–9. doi: 10.1089/bfm.2014.9986. PMID: 24823918 PMC4026103

[14] Zhang L Li Z Zhang B He H Bai Y . PPIA is a novel adipogenic factor implicated in obesity. Obes (Silver Spring). (2015) 23:2093–100. doi: 10.1002/oby.21208. PMID: 26347493

[15] Plows JF Stanley JL Baker PN Reynolds CM Vickers MH . The pathophysiology of gestational diabetes mellitus. Int J Mol Sci. (2018) 19:3342. doi: 10.3390/ijms19113342. PMID: 30373146 PMC6274679

[16] Usman TO Chhetri G Yeh H Dong HH . Beta-cell compensation and gestational diabetes. J Biol Chem. (2023) 299:105405. doi: 10.1016/j.jbc.2023.105405. PMID: 38229396 PMC10694657

[17] Cai Y Liu J Wang Q Ren X Xie J Yu J . Mechanisms of vascular endothelial cell injury triggered by blood glucose changes in gestational diabetes mellitus. Diabetes Obes Metab. (2025) 27:4203–19. doi: 10.1111/dom.16450. PMID: 40364503

[18] Nolan CJ Desoye G . Disentangling fetal insulin hypersecretion and insulin resistance. Trends Endocrinol Metab. (2024) 35:934–6. doi: 10.1016/j.tem.2024.04.008. PMID: 38697899

[19] Kalogeropoulou MS Couch H Thankamony A Beardsall K . Neonatal hyperinsulinism: a retrospective study of presentation and management in a tertiary neonatal intensive care unit in the UK. Arch Dis Child Fetal Neonatal Ed. (2025) 110:261–8. doi: 10.1136/archdischild-2024-327322. PMID: 39304222 PMC12013591

[20] Karcz K Królak-Olejnik B . Impact of gestational diabetes mellitus on fetal growth and nutritional status in newborns. Nutrients. (2024) 16:4093. doi: 10.3390/nu16234093. PMID: 39683486 PMC11643953

[21] Steiner A Rieder A Zaunschirm-Strutz J Weiss E Tocantins C Amtmann B . Umbilical cord plasma markers in gestational diabetes: evidence of fetal metaflammation, elevated GGT, and links to neonatal metabolic health. Am J Physiol Endocrinol Metab. (2026) 330:E369–79. doi: 10.1152/ajpendo.00404.2025. PMID: 41637729

[22] Li W Zhang S Liu H Wang L Zhang C Leng J . Different associations of diabetes with β-cell dysfunction and insulin resistance among obese and nonobese Chinese women with prior gestational diabetes mellitus. Diabetes Care. (2014) 37:2533–9. doi: 10.2337/dc14-0573. PMID: 24914241

[23] Iman AEH Onel C Furau G Furau C Furau R Lucan M . Evaluating the predictive value of HOMA-IR in gestational diabetes: a case-control study from Romania. Diagnostics (Basel). (2025) 15:1704. doi: 10.3390/diagnostics15131704. PMID: 40647703 PMC12248977

[24] Zhou X Zhang R Jiang S Cheng D Wu H . Analysis glycemic variability in pregnant women with various type of hyperglycemia. BMC Pregnancy Childbirth. (2025) 25:454. doi: 10.1186/s12884-025-07513-3. PMID: 40241083 PMC12004829

