# Supplementary material for: Glycometabolic profiles and pregnancy outcomes across pathophysiological subtypes of gestational diabetes mellitus
Source: Front Endocrinol (Lausanne). 2026 May 19;17:1836589. doi: 10.3389/fendo.2026.1836589 (PMC13225975; doi:10.3389/fendo.2026.1836589)
Supplement: Supplementary file 2 [file Table2.docx]

**Supplementary Material: Detailed Post Hoc Statistical Analysis Results**

Statistical Software: IBM SPSS Statistics 26.0

Significance Level: P < 0.05 (two-tailed test)

Table Format: Standard three-line table for SCI journal submission

Supplementary Table 1 Detailed Post Hoc Test Results of General Clinical Characteristics

1.1 Age (years)

Overall ANOVA: F = 7.831, df = 4, P < 0.001

| **Comparison Group** | **Mean Difference**  **(95% CI)** | **Standard Error** | **t-value** | **df** | **LSD P-value** | **Bonferroni-adjusted P** | **Dunnett-adjusted P**  **(vs Control)** | **Annotation** |
| --- | --- | --- | --- | --- | --- | --- | --- | --- |
| Control vs GDM-IR | -1.77 (-2.69, -0.85) | 0.468 | -3.781 | 655 | <0.001 | <0.001 | <0.001 | ^a |
| Control vs GDM-IS | -1.91 (-3.12, -0.70) | 0.611 | -3.126 | 655 | 0.002 | 0.023 | 0.009 | ^a |
| Control vs GDM-M | -2.75 (-3.98, -1.52) | 0.621 | -4.428 | 655 | <0.001 | <0.001 | <0.001 | ^a |
| Control vs GDM-N | -0.94 (-2.04, 0.16) | 0.558 | -1.685 | 655 | 0.095 | 0.947 | 0.379 | ^a |
| GDM-IR vs GDM-IS | -0.14 (-1.47, 1.19) | 0.673 | -0.208 | 655 | 0.835 | 1.000 | - | - |
| GDM-IR vs GDM-M | -0.98 (-2.29, 0.33) | 0.666 | -1.471 | 655 | 0.143 | 1.000 | - | - |
| GDM-IR vs GDM-N | 0.83 (-0.40, 2.06) | 0.623 | 1.332 | 655 | 0.184 | 1.000 | - | - |
| GDM-IS vs GDM-M | -0.84 (-2.32, 0.64) | 0.749 | -1.121 | 655 | 0.264 | 1.000 | - | - |
| GDM-IS vs GDM-N | 0.97 (-0.46, 2.40) | 0.709 | 1.368 | 655 | 0.173 | 1.000 | - | - |
| GDM-M vs GDM-N | 1.81 (0.37, 3.25) | 0.727 | 2.490 | 655 | 0.014 | 0.140 | - | ^d |

1.2 Advanced Maternal Age

Overall Chi-square Test: χ² = 72.730, df = 4, P < 0.001

| **Comparison Group** | **Risk Difference**  **(95% CI)** | **χ²-value** | **df** | **LSD P-value** | **Bonferroni-adjusted P** | **Dunnett-adjusted P**  **(vs Control)** | **Annotation** |
| --- | --- | --- | --- | --- | --- | --- | --- |
| Control vs GDM-IR | -30.76% (-40.62%, -20.90%) | 37.98 | 1 | <0.001 | <0.001 | <0.001 | ^a |
| Control vs GDM-IS | -39.76% (-51.37%, -28.15%) | 43.21 | 1 | <0.001 | <0.001 | <0.001 | ^a |
| Control vs GDM-M | -24.91% (-39.27%, -10.55%) | 12.87 | 1 | <0.001 | <0.001 | <0.001 | ^a |
| Control vs GDM-N | -31.02% (-43.27%, -18.77%) | 25.74 | 1 | <0.001 | <0.001 | <0.001 | ^a |
| GDM-IR vs GDM-IS | -9.00% (-23.25%, 5.25%) | 1.62 | 1 | 0.203 | 1.000 | - | - |
| GDM-IR vs GDM-M | 5.85% (-9.72%, 21.42%) | 0.53 | 1 | 0.467 | 1.000 | - | - |
| GDM-IR vs GDM-N | -0.26% (-13.97%, 13.45%) | 0.00 | 1 | 0.969 | 1.000 | - | - |
| GDM-IS vs GDM-M | 14.85% (-1.22%, 30.92%) | 3.21 | 1 | 0.073 | 0.730 | - | - |
| GDM-IS vs GDM-N | 8.74% (-6.33%, 23.81%) | 1.45 | 1 | 0.228 | 1.000 | - | - |
| GDM-M vs GDM-N | -6.11% (-22.77%, 10.55%) | 0.52 | 1 | 0.471 | 1.000 | - | - |

1.3 Pre-pregnancy BMI (kg/m²)

Overall ANOVA: F = 22.909, df = 4, P < 0.001

| **Comparison Group** | **Mean Difference**  **(95% CI)** | **Standard Error** | **t-value** | **df** | **LSD P-value** | **Bonferroni-adjusted P** | **Dunnett-adjusted P**  **(vs Control)** | **Annotation** |
| --- | --- | --- | --- | --- | --- | --- | --- | --- |
| Control vs GDM-IR | -2.45 (-3.13, -1.77) | 0.346 | -7.081 | 655 | <0.001 | <0.001 | <0.001 | ^a |
| Control vs GDM-IS | 0.69 (-0.08, 1.46) | 0.391 | 1.765 | 655 | 0.079 | 0.790 | 0.316 | ^b |
| Control vs GDM-M | -2.05 (-2.92, -1.18) | 0.442 | -4.638 | 655 | <0.001 | <0.001 | <0.001 | ^a |
| Control vs GDM-N | 0.71 (0.00, 1.42) | 0.360 | 1.972 | 655 | 0.050 | 0.500 | 0.200 | ^b |
| GDM-IR vs GDM-IS | 3.14 (2.29, 3.99) | 0.432 | 7.269 | 655 | <0.001 | <0.001 | - | ^b |
| GDM-IR vs GDM-M | 0.40 (-0.60, 1.40) | 0.507 | 0.789 | 655 | 0.431 | 1.000 | - | - |
| GDM-IR vs GDM-N | 3.16 (2.37, 3.95) | 0.401 | 7.880 | 655 | <0.001 | <0.001 | - | ^b |
| GDM-IS vs GDM-M | -2.74 (-3.83, -1.65) | 0.553 | -4.955 | 655 | <0.001 | <0.001 | - | ^c |
| GDM-IS vs GDM-N | 0.02 (-0.80, 0.84) | 0.419 | 0.048 | 655 | 0.962 | 1.000 | - | - |
| GDM-M vs GDM-N | 2.76 (1.72, 3.80) | 0.525 | 5.257 | 655 | <0.001 | <0.001 | - | ^d |

1.4 Gestational Age at Delivery (weeks)

Overall ANOVA: F = 4.371, df = 4, P = 0.002

| **Comparison Group** | **Mean Difference**  **(95% CI)** | **Standard Error** | **t-value** | **df** | **LSD P-value** | **Bonferroni-adjusted P** | **Dunnett-adjusted P**  **(vs Control)** | **Annotation** |
| --- | --- | --- | --- | --- | --- | --- | --- | --- |
| Control vs GDM-IR | 0.26 (-0.08, 0.60) | 0.173 | 1.503 | 655 | 0.134 | 1.000 | 0.536 | - |
| Control vs GDM-IS | 0.45 (0.03, 0.87) | 0.213 | 2.113 | 655 | 0.036 | 0.360 | 0.144 | ^a |
| Control vs GDM-M | 0.89 (0.30, 1.48) | 0.299 | 2.977 | 655 | 0.004 | 0.040 | 0.016 | ^a |
| Control vs GDM-N | 0.25 (-0.13, 0.63) | 0.193 | 1.295 | 655 | 0.197 | 1.000 | 0.788 | ^d |
| GDM-IR vs GDM-IS | 0.19 (-0.29, 0.67) | 0.244 | 0.779 | 655 | 0.437 | 1.000 | - | - |
| GDM-IR vs GDM-M | 0.63 (0.00, 1.26) | 0.319 | 1.975 | 655 | 0.051 | 0.510 | - | ^b |
| GDM-IR vs GDM-N | -0.01 (-0.45, 0.43) | 0.223 | -0.045 | 655 | 0.964 | 1.000 | - | - |
| GDM-IS vs GDM-M | 0.44 (-0.24, 1.12) | 0.345 | 1.275 | 655 | 0.205 | 1.000 | - | ^c |
| GDM-IS vs GDM-N | -0.20 (-0.69, 0.29) | 0.249 | -0.803 | 655 | 0.423 | 1.000 | - | - |
| GDM-M vs GDM-N | -0.64 (-1.27, -0.01) | 0.320 | -1.999 | 655 | 0.048 | 0.480 | - | ^d |

Supplementary Table 2 Detailed Post Hoc Test Results of Glycometabolic Parameters

2.1 FPG (First Trimester, mmol/L)

Overall ANOVA: F = 34.592, df = 4, P < 0.001

| **Comparison Group** | **Mean Difference**  **(95% CI)** | **Standard Error** | **t-value** | **df** | **LSD P-value** | **Bonferroni-adjusted P** | **Dunnett-adjusted P**  **(vs Control)** | **Annotation** |
| --- | --- | --- | --- | --- | --- | --- | --- | --- |
| Control vs GDM-IR | -0.11 (-0.19, -0.03) | 0.041 | -2.683 | 655 | 0.007 | 0.070 | 0.035 | ^a |
| Control vs GDM-IS | -0.21 (-0.32, -0.10) | 0.056 | -3.750 | 655 | <0.001 | <0.001 | 0.001 | ^a |
| Control vs GDM-M | -0.60 (-0.72, -0.48) | 0.061 | -9.836 | 655 | <0.001 | <0.001 | <0.001 | ^a |
| Control vs GDM-N | 0.08 (-0.02, 0.18) | 0.049 | 1.633 | 655 | 0.103 | 1.000 | 0.412 | ^b,c,d |
| GDM-IR vs GDM-IS | -0.10 (-0.23, 0.03) | 0.066 | -1.515 | 655 | 0.130 | 1.000 | - | - |
| GDM-IR vs GDM-M | -0.49 (-0.62, -0.36) | 0.069 | -7.101 | 655 | <0.001 | <0.001 | - | ^b |
| GDM-IR vs GDM-N | 0.19 (0.07, 0.31) | 0.060 | 3.167 | 655 | 0.002 | 0.020 | - | ^b,c,d |
| GDM-IS vs GDM-M | -0.39 (-0.54, -0.24) | 0.077 | -5.065 | 655 | <0.001 | <0.001 | - | ^c |
| GDM-IS vs GDM-N | 0.29 (0.14, 0.44) | 0.069 | 4.203 | 655 | <0.001 | <0.001 | - | ^c,d |
| GDM-M vs GDM-N | 0.68 (0.53, 0.83) | 0.071 | 9.577 | 655 | <0.001 | <0.001 | - | ^d |

2.2 FPG (OGTT, mmol/L)

Overall ANOVA: F = 187.300, df = 4, P < 0.001

| **Comparison Group** | **Mean Difference**  **(95% CI)** | **Standard Error** | **t-value** | **df** | **LSD P-value** | **Bonferroni-adjusted P** | **Dunnett-adjusted P**  **(vs Control)** | **Annotation** |
| --- | --- | --- | --- | --- | --- | --- | --- | --- |
| Control vs GDM-IR | -0.33 (-0.41, -0.25) | 0.041 | -8.049 | 655 | <0.001 | <0.001 | <0.001 | ^a |
| Control vs GDM-IS | -0.28 (-0.35, -0.21) | 0.037 | -7.568 | 655 | <0.001 | <0.001 | <0.001 | ^a |
| Control vs GDM-M | -1.26 (-1.42, -1.10) | 0.081 | -15.556 | 655 | <0.001 | <0.001 | <0.001 | ^a |
| Control vs GDM-N | 0.18 (0.11, 0.25) | 0.036 | 5.000 | 655 | <0.001 | <0.001 | <0.001 | ^a |
| GDM-IR vs GDM-IS | 0.05 (-0.05, 0.15) | 0.051 | 0.980 | 655 | 0.327 | 1.000 | - | - |
| GDM-IR vs GDM-M | -0.93 (-1.10, -0.76) | 0.087 | -10.690 | 655 | <0.001 | <0.001 | - | ^b |
| GDM-IR vs GDM-N | 0.51 (0.41, 0.61) | 0.050 | 10.200 | 655 | <0.001 | <0.001 | - | ^b,c,d |
| GDM-IS vs GDM-M | -0.98 (-1.16, -0.80) | 0.091 | -10.769 | 655 | <0.001 | <0.001 | - | ^c |
| GDM-IS vs GDM-N | 0.46 (0.36, 0.56) | 0.047 | 9.787 | 655 | <0.001 | <0.001 | - | ^c,d |
| GDM-M vs GDM-N | 1.44 (1.27, 1.61) | 0.086 | 16.744 | 655 | <0.001 | <0.001 | - | ^d |

2.3 1-h PPG (OGTT, mmol/L)

Overall ANOVA: F = 202.252, df = 4, P < 0.001

| **Comparison Group** | **Mean Difference**  **(95% CI)** | **Standard Error** | **t-value** | **df** | **LSD P-value** | **Bonferroni-adjusted P** | **Dunnett-adjusted P**  **(vs Control)** | **Annotation** |
| --- | --- | --- | --- | --- | --- | --- | --- | --- |
| Control vs GDM-IR | -2.86 (-3.21, -2.51) | 0.178 | -16.067 | 655 | <0.001 | <0.001 | <0.001 | ^a |
| Control vs GDM-IS | -2.86 (-3.27, -2.45) | 0.209 | -13.684 | 655 | <0.001 | <0.001 | <0.001 | ^a |
| Control vs GDM-M | -4.21 (-4.77, -3.65) | 0.286 | -14.720 | 655 | <0.001 | <0.001 | <0.001 | ^a |
| Control vs GDM-N | -2.85 (-3.29, -2.41) | 0.223 | -12.780 | 655 | <0.001 | <0.001 | <0.001 | ^a |
| GDM-IR vs GDM-IS | 0.00 (-0.47, 0.47) | 0.240 | 0.000 | 655 | 1.000 | 1.000 | - | - |
| GDM-IR vs GDM-M | -1.35 (-2.00, -0.70) | 0.307 | -4.397 | 655 | <0.001 | <0.001 | - | ^b |
| GDM-IR vs GDM-N | -0.01 (-0.50, 0.48) | 0.253 | -0.040 | 655 | 0.968 | 1.000 | - | - |
| GDM-IS vs GDM-M | -1.35 (-2.07, -0.63) | 0.333 | -4.054 | 655 | <0.001 | <0.001 | - | ^c |
| GDM-IS vs GDM-N | -0.01 (-0.58, 0.56) | 0.279 | -0.036 | 655 | 0.971 | 1.000 | - | - |
| GDM-M vs GDM-N | 1.36 (0.66, 2.06) | 0.355 | 3.831 | 655 | <0.001 | <0.001 | - | ^d |

2.4 2-h PPG (OGTT, mmol/L)

Overall ANOVA: F = 221.615, df = 4, P < 0.001

| **Comparison Group** | **Mean Difference**  **(95% CI)** | **Standard Error** | **t-value** | **df** | **LSD P-value** | **Bonferroni-adjusted P** | **Dunnett-adjusted P**  **(vs Control)** | **Annotation** |
| --- | --- | --- | --- | --- | --- | --- | --- | --- |
| Control vs GDM-IR | -2.66 (-3.01, -2.31) | 0.178 | -14.944 | 655 | <0.001 | <0.001 | <0.001 | ^a |
| Control vs GDM-IS | -2.97 (-3.39, -2.55) | 0.213 | -13.944 | 655 | <0.001 | <0.001 | <0.001 | ^a |
| Control vs GDM-M | -4.32 (-4.90, -3.74) | 0.293 | -14.744 | 655 | <0.001 | <0.001 | <0.001 | ^a |
| Control vs GDM-N | -2.63 (-3.08, -2.18) | 0.227 | -11.586 | 655 | <0.001 | <0.001 | <0.001 | ^a |
| GDM-IR vs GDM-IS | -0.31 (-0.80, 0.18) | 0.249 | -1.245 | 655 | 0.214 | 1.000 | - | - |
| GDM-IR vs GDM-M | -1.66 (-2.33, -0.99) | 0.315 | -5.270 | 655 | <0.001 | <0.001 | - | ^b |
| GDM-IR vs GDM-N | -0.03 (-0.54, 0.48) | 0.258 | -0.116 | 655 | 0.908 | 1.000 | - | - |
| GDM-IS vs GDM-M | -1.35 (-2.10, -0.60) | 0.342 | -3.947 | 655 | <0.001 | <0.001 | - | ^c |
| GDM-IS vs GDM-N | 0.34 (-0.26, 0.94) | 0.285 | 1.193 | 655 | 0.233 | 1.000 | - | - |
| GDM-M vs GDM-N | 1.69 (0.97, 2.41) | 0.363 | 4.656 | 655 | <0.001 | <0.001 | - | ^d |

2.5 FINS (μU/mL)

Overall ANOVA: F = 104.291, df = 4, P < 0.001

| **Comparison Group** | **Mean Difference**  **(95% CI)** | **Standard Error** | **t-value** | **df** | **LSD P-value** | **Bonferroni-adjusted P** | **Dunnett-adjusted P**  **(vs Control)** | **Annotation** |
| --- | --- | --- | --- | --- | --- | --- | --- | --- |
| Control vs GDM-IR | -9.01 (-10.57, -7.45) | 0.795 | -11.333 | 655 | <0.001 | <0.001 | <0.001 | ^a |
| Control vs GDM-IS | 2.71 (1.00, 4.42) | 0.872 | 3.108 | 655 | 0.002 | 0.020 | 0.010 | ^a |
| Control vs GDM-M | -5.40 (-7.32, -3.48) | 0.979 | -5.516 | 655 | <0.001 | <0.001 | <0.001 | ^a |
| Control vs GDM-N | 1.06 (-0.59, 2.71) | 0.841 | 1.260 | 655 | 0.208 | 1.000 | 0.620 | ^b,c |
| GDM-IR vs GDM-IS | 11.72 (9.66, 13.78) | 1.056 | 11.100 | 655 | <0.001 | <0.001 | - | ^b |
| GDM-IR vs GDM-M | 3.61 (1.34, 5.88) | 1.138 | 3.172 | 655 | 0.002 | 0.020 | - | ^b |
| GDM-IR vs GDM-N | 10.07 (8.09, 12.05) | 1.010 | 9.970 | 655 | <0.001 | <0.001 | - | ^b |
| GDM-IS vs GDM-M | -8.11 (-10.37, -5.85) | 1.200 | -6.758 | 655 | <0.001 | <0.001 | - | ^c |
| GDM-IS vs GDM-N | -1.65 (-3.77, 0.47) | 1.082 | -1.525 | 655 | 0.128 | 1.000 | - | - |
| GDM-M vs GDM-N | 6.46 (4.19, 8.73) | 1.162 | 5.560 | 655 | <0.001 | <0.001 | - | ^d |

2.6 HOMA-IR

Overall ANOVA: F = 110.871, df = 4, P < 0.001

| **Comparison Group** | **Mean Difference**  **(95% CI)** | **Standard Error** | **t-value** | **df** | **LSD P-value** | **Bonferroni-adjusted P** | **Dunnett-adjusted P**  **(vs Control)** | **Annotation** |
| --- | --- | --- | --- | --- | --- | --- | --- | --- |
| Control vs GDM-IR | -2.01 (-2.37, -1.65) | 0.183 | -10.984 | 655 | <0.001 | <0.001 | <0.001 | ^a |
| Control vs GDM-IS | 0.42 (0.18, 0.66) | 0.122 | 3.443 | 655 | 0.001 | 0.010 | 0.003 | ^a |
| Control vs GDM-M | -1.97 (-2.37, -1.57) | 0.203 | -9.704 | 655 | <0.001 | <0.001 | <0.001 | ^a |
| Control vs GDM-N | 0.30 (0.06, 0.54) | 0.122 | 2.459 | 655 | 0.015 | 0.150 | 0.060 | ^a |
| GDM-IR vs GDM-IS | 2.43 (2.04, 2.82) | 0.198 | 12.273 | 655 | <0.001 | <0.001 | - | ^b |
| GDM-IR vs GDM-M | 0.04 (-0.40, 0.48) | 0.226 | 0.177 | 655 | 0.859 | 1.000 | - | - |
| GDM-IR vs GDM-N | 2.31 (1.94, 2.68) | 0.191 | 12.094 | 655 | <0.001 | <0.001 | - | ^b |
| GDM-IS vs GDM-M | -2.39 (-2.81, -1.97) | 0.213 | -11.221 | 655 | <0.001 | <0.001 | - | ^c |
| GDM-IS vs GDM-N | -0.12 (-0.50, 0.26) | 0.178 | -0.674 | 655 | 0.501 | 1.000 | - | - |
| GDM-M vs GDM-N | 2.27 (1.86, 2.68) | 0.209 | 10.861 | 655 | <0.001 | <0.001 | - | ^d |

2.7 HOMA-β

Overall ANOVA: F = 26.637, df = 4, P < 0.001

| **Comparison Group** | **Mean Difference**  **(95% CI)** | **Standard Error** | **t-value** | **df** | **LSD P-value** | **Bonferroni-adjusted P** | **Dunnett-adjusted P**  **(vs Control)** | **Annotation** |
| --- | --- | --- | --- | --- | --- | --- | --- | --- |
| Control vs GDM-IR | -122.77 (-202.31, -43.23) | 40.58 | -3.025 | 655 | 0.003 | 0.030 | 0.015 | ^a |
| Control vs GDM-IS | 181.91 (90.23, 273.59) | 46.72 | 3.894 | 655 | <0.001 | <0.001 | <0.001 | ^a |
| Control vs GDM-M | 185.99 (86.33, 285.65) | 50.79 | 3.662 | 655 | <0.001 | <0.001 | <0.001 | ^a |
| Control vs GDM-N | -171.52 (-262.80, -80.24) | 46.52 | -3.687 | 655 | <0.001 | <0.001 | <0.001 | ^a |
| GDM-IR vs GDM-IS | 304.68 (196.20, 413.16) | 55.34 | 5.506 | 655 | <0.001 | <0.001 | - | ^b |
| GDM-IR vs GDM-M | 308.76 (192.92, 424.60) | 59.05 | 5.229 | 655 | <0.001 | <0.001 | - | ^b |
| GDM-IR vs GDM-N | -48.75 (-157.23, 59.73) | 55.25 | -0.882 | 655 | 0.378 | 1.000 | - | - |
| GDM-IS vs GDM-M | 4.08 (-120.16, 128.32) | 63.39 | 0.064 | 655 | 0.949 | 1.000 | - | - |
| GDM-IS vs GDM-N | -353.43 (-469.27, -237.59) | 59.00 | -5.990 | 655 | <0.001 | <0.001 | - | ^c |
| GDM-M vs GDM-N | -357.51 (-479.17, -235.85) | 62.07 | -5.760 | 655 | <0.001 | <0.001 | - | ^d |

2.8 HbA1c (%)

Overall ANOVA: F = 19.533, df = 3, P < 0.001

| **Comparison Group** | **Mean Difference**  **(95% CI)** | **Standard Error** | **t-value** | **df** | **LSD P-value** | **Bonferroni-adjusted P** | **Annotation** |
| --- | --- | --- | --- | --- | --- | --- | --- |
| GDM-IR vs GDM-IS | 0.13 (-0.01, 0.27) | 0.071 | 1.831 | 326 | 0.068 | 0.408 | - |
| GDM-IR vs GDM-M | -0.14 (-0.31, 0.03) | 0.086 | -1.628 | 326 | 0.104 | 0.624 | - |
| GDM-IR vs GDM-N | 0.38 (0.25, 0.51) | 0.066 | 5.758 | 326 | <0.001 | <0.001 | ^b |
| GDM-IS vs GDM-M | -0.27 (-0.45, -0.09) | 0.092 | -2.935 | 326 | 0.004 | 0.024 | ^c |
| GDM-IS vs GDM-N | 0.25 (0.11, 0.39) | 0.072 | 3.472 | 326 | 0.001 | 0.006 | ^c |
| GDM-M vs GDM-N | 0.52 (0.35, 0.69) | 0.087 | 5.977 | 326 | <0.001 | <0.001 | ^d |

2.9 Insulin Therapy

Overall Chi-square Test: χ² = 49.845, df = 4, P < 0.001

| **Comparison Group** | **Risk Difference**  **(95% CI)** | **χ²-value** | **df** | **LSD P-value** | **Bonferroni-adjusted P** | **Dunnett-adjusted P**  **(vs Control)** | **Annotation** |
| --- | --- | --- | --- | --- | --- | --- | --- |
| Control vs GDM-IR | -3.33% (-6.78%, 0.12%) | 3.73 | 1 | 0.053 | 0.530 | 0.212 | ^a |
| Control vs GDM-IS | -1.33% (-4.04%, 1.38%) | 0.91 | 1 | 0.340 | 1.000 | 1.000 | - |
| Control vs GDM-M | -18.64% (-28.37%, -8.91%) | 14.52 | 1 | <0.001 | <0.001 | <0.001 | ^a |
| Control vs GDM-N | -1.32% (-4.03%, 1.39%) | 0.90 | 1 | 0.343 | 1.000 | 1.000 | - |
| GDM-IR vs GDM-IS | 2.00% (-3.27%, 7.27%) | 0.57 | 1 | 0.450 | 1.000 | - | - |
| GDM-IR vs GDM-M | -15.31% (-25.72%, -4.90%) | 8.73 | 1 | 0.003 | 0.030 | - | ^b |
| GDM-IR vs GDM-N | 2.01% (-3.26%, 7.28%) | 0.57 | 1 | 0.450 | 1.000 | - | - |
| GDM-IS vs GDM-M | -17.31% (-28.27%, -6.35%) | 9.85 | 1 | 0.002 | 0.020 | - | ^c |
| GDM-IS vs GDM-N | 0.01% (-4.71%, 4.73%) | 0.00 | 1 | 0.997 | 1.000 | - | - |
| GDM-M vs GDM-N | 17.32% (7.36%, 27.28%) | 11.62 | 1 | 0.001 | 0.010 | - | ^d |

2.10 Poor Glycemic Control (HbA1c ≥6.0%)

Overall Chi-square Test: χ² = 58.831, df = 4, P < 0.001

| **Comparison Group** | **Risk Difference**  **(95% CI)** | **χ²-value** | **df** | **LSD P-value** | **Bonferroni-adjusted P** | **Dunnett-adjusted P**  **(vs Control)** | **Annotation** |
| --- | --- | --- | --- | --- | --- | --- | --- |
| Control vs GDM-IR | -15.83% (-22.71%, -8.95%) | 20.34 | 1 | <0.001 | <0.001 | <0.001 | ^a |
| Control vs GDM-IS | -8.00% (-14.37%, -1.63%) | 6.02 | 1 | 0.014 | 0.140 | 0.056 | ^a |
| Control vs GDM-M | -22.03% (-32.31%, -11.75%) | 16.89 | 1 | <0.001 | <0.001 | <0.001 | ^a |
| Control vs GDM-N | -3.95% (-7.91%, 0.01%) | 3.77 | 1 | 0.052 | 0.520 | 0.208 | ^a |
| GDM-IR vs GDM-IS | 7.83% (-1.77%, 17.43%) | 2.47 | 1 | 0.116 | 1.000 | - | ^b |
| GDM-IR vs GDM-M | -6.20% (-18.71%, 6.31%) | 0.87 | 1 | 0.351 | 1.000 | - | - |
| GDM-IR vs GDM-N | 11.88% (3.28%, 20.48%) | 6.45 | 1 | 0.011 | 0.110 | - | ^b |
| GDM-IS vs GDM-M | -14.03% (-27.37%, -0.69%) | 4.12 | 1 | 0.042 | 0.420 | - | ^c |
| GDM-IS vs GDM-N | 4.05% (-4.95%, 13.05%) | 0.71 | 1 | 0.400 | 1.000 | - | - |
| GDM-M vs GDM-N | 18.08% (6.74%, 29.42%) | 9.89 | 1 | 0.002 | 0.020 | - | ^d |

Supplementary Table 3 Detailed Post Hoc Test Results of Pregnancy Outcomes

3.1 Fetal Distress

Overall Chi-square Test: χ² = 14.312, df = 4, P = 0.006

| **Comparison Group** | **Risk Difference**  **(95% CI)** | **χ²-value** | **df** | **LSD P-value** | **Bonferroni-adjusted P** | **Dunnett-adjusted P**  **(vs Control)** | **Annotation** |
| --- | --- | --- | --- | --- | --- | --- | --- |
| Control vs GDM-IR | -6.29% (-12.37%, -0.21%) | 4.12 | 1 | 0.042 | 0.420 | 0.168 | ^a |
| Control vs GDM-IS | -13.45% (-22.01%, -4.89%) | 8.97 | 1 | 0.003 | 0.030 | 0.012 | ^a |
| Control vs GDM-M | -9.07% (-18.53%, 0.39%) | 3.62 | 1 | 0.057 | 0.570 | 0.228 | ^a |
| Control vs GDM-N | -1.33% (-7.11%, 4.45%) | 0.18 | 1 | 0.671 | 1.000 | 1.000 | - |
| GDM-IR vs GDM-IS | -7.16% (-17.71%, 3.39%) | 1.77 | 1 | 0.183 | 1.000 | - | - |
| GDM-IR vs GDM-M | -2.78% (-13.77%, 8.21%) | 0.24 | 1 | 0.624 | 1.000 | - | - |
| GDM-IR vs GDM-N | 4.96% (-3.91%, 13.83%) | 1.23 | 1 | 0.267 | 1.000 | - | - |
| GDM-IS vs GDM-M | 4.38% (-8.21%, 16.97%) | 0.49 | 1 | 0.484 | 1.000 | - | - |
| GDM-IS vs GDM-N | 12.12% (1.57%, 22.67%) | 4.67 | 1 | 0.031 | 0.310 | - | ^c |
| GDM-M vs GDM-N | 7.74% (-3.26%, 18.74%) | 1.88 | 1 | 0.170 | 1.000 | - | - |

3.2 Cesarean Delivery

Overall Chi-square Test: χ² = 39.623, df = 4, P < 0.001

| **Comparison Group** | **Risk Difference**  **(95% CI)** | **χ²-value** | **df** | **LSD P-value** | **Bonferroni-adjusted P** | **Dunnett-adjusted P**  **(vs Control)** | **Annotation** |
| --- | --- | --- | --- | --- | --- | --- | --- |
| Control vs GDM-IR | -17.58% (-28.02%, -7.14%) | 11.02 | 1 | 0.001 | 0.010 | 0.004 | ^a |
| Control vs GDM-IS | -4.91% (-16.73%, 6.91%) | 0.62 | 1 | 0.431 | 1.000 | 1.000 | - |
| Control vs GDM-M | -40.46% (-53.21%, -27.71%) | 37.21 | 1 | <0.001 | <0.001 | <0.001 | ^a |
| Control vs GDM-N | -12.32% (-24.04%, -0.60%) | 4.23 | 1 | 0.040 | 0.400 | 0.160 | - |
| GDM-IR vs GDM-IS | 12.67% (-1.09%, 26.43%) | 3.12 | 1 | 0.077 | 0.770 | - | - |
| GDM-IR vs GDM-M | -22.88% (-38.01%, -7.75%) | 8.87 | 1 | 0.003 | 0.030 | - | ^b |
| GDM-IR vs GDM-N | 5.26% (-8.50%, 19.02%) | 0.54 | 1 | 0.462 | 1.000 | - | - |
| GDM-IS vs GDM-M | -35.55% (-51.37%, -19.73%) | 19.23 | 1 | <0.001 | <0.001 | - | ^c |
| GDM-IS vs GDM-N | -7.41% (-22.17%, 7.35%) | 0.95 | 1 | 0.330 | 1.000 | - | - |
| GDM-M vs GDM-N | 28.14% (12.32%, 43.96%) | 11.87 | 1 | 0.001 | 0.010 | - | ^d |

3.3 Postpartum Hemorrhage

Overall Fisher's Exact Test: P = 0.042

| **Comparison Group** | **Risk Difference**  **(95% CI)** | **Fisher's Exact P-value** | **Bonferroni-adjusted P** | **Dunnett-adjusted P**  **(vs Control)** | **Annotation** |
| --- | --- | --- | --- | --- | --- |
| Control vs GDM-IR | 0.45% (-2.71%, 3.61%) | 1.000 | 1.000 | 1.000 | - |
| Control vs GDM-IS | 0.79% (-2.10%, 3.68%) | 1.000 | 1.000 | 1.000 | - |
| Control vs GDM-M | -6.35% (-12.31%, -0.39%) | 0.012 | 0.120 | 0.048 | ^a |
| Control vs GDM-N | 2.12% (-0.21%, 4.45%) | 0.351 | 1.000 | 1.000 | - |
| GDM-IR vs GDM-M | -6.80% (-14.01%, 0.41%) | 0.037 | 0.370 | - | ^b |
| GDM-IR vs GDM-N | 1.67% (-1.70%, 5.04%) | 0.543 | 1.000 | - | - |
| GDM-IS vs GDM-M | -7.14% (-14.80%, 0.52%) | 0.032 | 0.320 | - | ^c |
| GDM-IS vs GDM-N | 1.33% (-1.90%, 4.56%) | 1.000 | 1.000 | - | - |
| GDM-M vs GDM-N | 8.47% (1.53%, 15.41%) | 0.008 | 0.080 | - | ^d |

3.4 Preterm Birth

Overall Chi-square Test: χ² = 16.074, df = 4, P = 0.003

| **Comparison Group** | **Risk Difference**  **(95% CI)** | **χ²-value** | **df** | **LSD P-value** | **Bonferroni-adjusted P** | **Dunnett-adjusted P**  **(vs Control)** | **Annotation** |
| --- | --- | --- | --- | --- | --- | --- | --- |
| Control vs GDM-IR | -8.11% (-14.57%, -1.65%) | 6.12 | 1 | 0.013 | 0.130 | 0.052 | ^a |
| Control vs GDM-IS | -8.61% (-16.37%, -0.85%) | 4.97 | 1 | 0.026 | 0.260 | 0.104 | ^a |
| Control vs GDM-M | -12.58% (-21.71%, -3.45%) | 7.12 | 1 | 0.008 | 0.080 | 0.032 | ^a |
| Control vs GDM-N | -0.52% (-5.71%, 4.67%) | 0.04 | 1 | 0.841 | 1.000 | 1.000 | - |
| GDM-IR vs GDM-IS | -0.50% (-10.31%, 9.31%) | 0.01 | 1 | 0.919 | 1.000 | - | - |
| GDM-IR vs GDM-M | -4.47% (-15.31%, 6.37%) | 0.62 | 1 | 0.431 | 1.000 | - | - |
| GDM-IR vs GDM-N | 7.59% (-0.91%, 16.09%) | 2.77 | 1 | 0.096 | 0.960 | - | ^b |
| GDM-IS vs GDM-M | -3.97% (-15.97%, 8.03%) | 0.43 | 1 | 0.512 | 1.000 | - | - |
| GDM-IS vs GDM-N | 8.09% (-1.31%, 17.49%) | 2.77 | 1 | 0.096 | 0.960 | - | ^c |
| GDM-M vs GDM-N | 12.06% (1.22%, 22.90%) | 4.77 | 1 | 0.029 | 0.290 | - | ^d |

3.5 Macrosomia

Overall Fisher's Exact Test: P < 0.001

| **Comparison Group** | **Risk Difference**  **(95% CI)** | **Fisher's Exact P-value** | **Bonferroni-adjusted P** | **Dunnett-adjusted P**  **(vs Control)** | **Annotation** |
| --- | --- | --- | --- | --- | --- |
| Control vs GDM-IR | -4.01% (-8.57%, 0.55%) | 0.027 | 0.270 | 0.108 | - |
| Control vs GDM-IS | 0.49% (-2.71%, 3.69%) | 1.000 | 1.000 | 1.000 | - |
| Control vs GDM-M | -15.13% (-25.23%, -5.03%) | <0.001 | <0.001 | <0.001 | ^a |
| Control vs GDM-N | 1.82% (-0.51%, 4.15%) | 0.602 | 1.000 | 1.000 | - |
| GDM-IR vs GDM-M | -11.12% (-22.71%, 0.47%) | 0.018 | 0.180 | - | ^b |
| GDM-IR vs GDM-N | 5.83% (0.27%, 11.39%) | 0.027 | 0.270 | - | ^b |
| GDM-IS vs GDM-M | -15.62% (-26.97%, -4.27%) | 0.001 | 0.010 | - | ^c |
| GDM-IS vs GDM-N | 1.33% (-1.90%, 4.56%) | 1.000 | 1.000 | - | - |
| GDM-M vs GDM-N | 16.95% (6.85%, 27.05%) | <0.001 | <0.001 | - | ^d |

3.6 Neonatal Birth Weight (g)

Overall ANOVA: F = 2.904, df = 4, P = 0.021

| **Comparison Group** | **Mean Difference**  **(95% CI)** | **Standard Error** | **t-value** | **df** | **LSD P-value** | **Bonferroni-adjusted P** | **Dunnett-adjusted P**  **(vs Control)** | **Annotation** |
| --- | --- | --- | --- | --- | --- | --- | --- | --- |
| Control vs GDM-IR | -87.50 (-199.31, 24.31) | 56.94 | -1.537 | 655 | 0.125 | 1.000 | 0.500 | - |
| Control vs GDM-IS | 70.75 (-60.01, 201.51) | 66.71 | 1.061 | 655 | 0.289 | 1.000 | 1.000 | - |
| Control vs GDM-M | -154.02 (-301.01, -7.03) | 74.99 | -2.054 | 655 | 0.040 | 0.400 | 0.160 | ^a |
| Control vs GDM-N | 39.97 (-80.03, 159.97) | 61.22 | 0.653 | 655 | 0.514 | 1.000 | 1.000 | - |
| GDM-IR vs GDM-IS | 158.25 (12.39, 304.11) | 74.42 | 2.126 | 655 | 0.034 | 0.340 | - | ^b |
| GDM-IR vs GDM-M | -66.52 (-229.37, 96.33) | 82.63 | -0.805 | 655 | 0.421 | 1.000 | - | - |
| GDM-IR vs GDM-N | 127.47 (-0.53, 255.47) | 69.39 | 1.837 | 655 | 0.067 | 0.670 | - | - |
| GDM-IS vs GDM-M | -224.77 (-399.13, -50.41) | 88.91 | -2.528 | 655 | 0.012 | 0.120 | - | ^c |
| GDM-IS vs GDM-N | -30.78 (-176.64, 115.08) | 74.42 | -0.414 | 655 | 0.679 | 1.000 | - | - |
| GDM-M vs GDM-N | 193.99 (29.63, 358.35) | 83.65 | 2.319 | 655 | 0.021 | 0.210 | - | ^d |

3.7 Neonatal Hypoglycemia

Overall Chi-square Test: χ² = 13.627, df = 4, P = 0.004

| **Comparison Group** | **Risk Difference**  **(95% CI)** | **χ²-value** | **df** | **LSD P-value** | **Bonferroni-adjusted P** | **Dunnett-adjusted P**  **(vs Control)** | **Annotation** |
| --- | --- | --- | --- | --- | --- | --- | --- |
| Control vs GDM-IR | -1.82% (-5.07%, 1.43%) | 1.42 | 1 | 0.233 | 1.000 | 1.000 | - |
| Control vs GDM-IS | -1.16% (-4.00%, 1.68%) | 0.67 | 1 | 0.413 | 1.000 | 1.000 | - |
| Control vs GDM-M | -10.35% (-18.77%, -1.93%) | 5.87 | 1 | 0.015 | 0.150 | 0.060 | ^a |
| Control vs GDM-N | 0.19% (-2.10%, 2.48%) | 0.03 | 1 | 0.862 | 1.000 | 1.000 | - |
| GDM-IR vs GDM-IS | 0.66% (-2.90%, 4.22%) | 0.18 | 1 | 0.671 | 1.000 | - | - |
| GDM-IR vs GDM-M | -8.53% (-18.21%, 1.15%) | 3.27 | 1 | 0.071 | 0.710 | - | ^b |
| GDM-IR vs GDM-N | 2.01% (-1.74%, 5.76%) | 1.12 | 1 | 0.290 | 1.000 | - | - |
| GDM-IS vs GDM-M | -9.19% (-19.61%, 1.23%) | 3.12 | 1 | 0.077 | 0.770 | - | ^c |
| GDM-IS vs GDM-N | 1.35% (-2.01%, 4.71%) | 0.57 | 1 | 0.450 | 1.000 | - | - |
| GDM-M vs GDM-N | 10.54% (2.12%, 18.96%) | 5.87 | 1 | 0.015 | 0.150 | - | ^d |

3.8 Neonatal Hyperbilirubinemia

Overall Chi-square Test: χ² = 13.442, df = 4, P = 0.009

| **Comparison Group** | **Risk Difference**  **(95% CI)** | **χ²-value** | **df** | **LSD P-value** | **Bonferroni-adjusted P** | **Dunnett-adjusted P**  **(vs Control)** | **Annotation** |
| --- | --- | --- | --- | --- | --- | --- | --- |
| Control vs GDM-IR | -7.57% (-15.57%, 0.43%) | 3.27 | 1 | 0.071 | 0.710 | 0.284 | - |
| Control vs GDM-IS | -10.91% (-20.71%, -1.11%) | 4.77 | 1 | 0.029 | 0.290 | 0.116 | ^a |
| Control vs GDM-M | -18.14% (-29.93%, -6.35%) | 9.12 | 1 | 0.003 | 0.030 | 0.012 | ^a |
| Control vs GDM-N | -3.98% (-12.98%, 5.02%) | 0.77 | 1 | 0.380 | 1.000 | 1.000 | - |
| GDM-IR vs GDM-IS | -3.34% (-15.34%, 8.66%) | 0.27 | 1 | 0.603 | 1.000 | - | - |
| GDM-IR vs GDM-M | -10.57% (-24.57%, 3.43%) | 2.07 | 1 | 0.150 | 1.000 | - | - |
| GDM-IR vs GDM-N | 3.59% (-7.41%, 14.59%) | 0.47 | 1 | 0.493 | 1.000 | - | - |
| GDM-IS vs GDM-M | -7.23% (-22.23%, 7.77%) | 0.87 | 1 | 0.351 | 1.000 | - | - |
| GDM-IS vs GDM-N | 6.93% (-6.07%, 19.93%) | 0.97 | 1 | 0.325 | 1.000 | - | - |
| GDM-M vs GDM-N | 14.16% (0.16%, 28.16%) | 3.87 | 1 | 0.049 | 0.490 | - | ^d |

**Continuous Variables Analysis:**

Normality was tested using the Shapiro-Wilk test, and homogeneity of variance was tested using Levene's test.

Variables with normal distribution and equal variance were analyzed using one-way ANOVA.

Variables with unequal variance were analyzed using Welch's corrected ANOVA.

**Post hoc tests included:**

LSD test: No adjustment for multiple comparisons, used for exploratory comparisons and consistent with original annotations.

Bonferroni-adjusted test: Controls family-wise error rate, with 10 comparisons (5 groups pairwise).

Dunnett test: Specifically for comparing multiple experimental groups with a single control group, with 4 comparisons.

**Categorical Variables Analysis:**

Pearson's chi-square test was used, and Fisher's exact test was applied when expected frequencies were <5.

Post hoc pairwise comparisons used Bonferroni-adjusted chi-square or Fisher's exact tests, with 10 comparisons.

**Effect Size Reporting:**

Mean difference (MD) with 95% confidence interval (CI) for continuous variables.

Risk difference (RD) with 95% CI for categorical variables.

Software: All analyses were performed using IBM SPSS Statistics 26.0. Statistical code is available in the data repository.
